# Evaluation of CD3 and CD20 Lymphocytes and Mast Cells in the Microenvironment of Central Giant Cell Granuloma, Peripheral Giant Cell Granuloma, and Giant Cell Tumor of Bone

**DOI:** 10.3390/diagnostics16010090

**Published:** 2025-12-26

**Authors:** Khelan Ahmed Hama Faeq, Balkees T. Gharib

**Affiliations:** Department of Oral Pathology, College of Dentistry, University of Suleimani, Sulaymaniyah 46001, Iraq; balkees.garib@univsul.edu.iq

**Keywords:** central giant cell granuloma, peripheral giant cell granuloma, giant cell tumor of bones, mast cell, lymphocyte

## Abstract

**Objective**: Giant cell lesions (GCLs) share similar histopathologic features. The influence of immune involvement on the biology of giant cell lesions remains largely elusive. This study aimed to evaluate and compare lymphocyte and mast cell infiltration and distribution among three giant cell lesions. **Study design**: A total of 30 FFPE tissue blocks, comprising 10 PGCGs, 10 CGCGs (aggressive and nonaggressive), and 10 GCTs (aggressive and nonaggressive) of bone, were subjected to IHC staining for CD3 and CD20 lymphocyte markers and toluidine blue staining for mast cells. The mean count of positively stained cells was calculated and categorized into three scores, along with a group for negative cases. Statistical analysis was conducted to assess significance at *p* < 0.05. **Result**: Lymphocyte infiltration was observed across all lesions. CD3^+^ and CD20^+^ cell counts were significantly elevated in PGCGs, followed by CGCGs, and were lowest in GCTs of bone. In contrast, mast cell counts were high in GCTs of bone and CGCGs and low in PGCGs. Aggressive giant cell lesions of bone showed a significantly low number of CD3^+^ and CD20^+^ cells (Mann–Whitney U test; *p* = 0.05, 0.004) and a high number of mast cells (Mann–Whitney U test; *p* < 0.001) compared with nonaggressive lesions of bone. PGCGs and nonaggressive CGCGs showed comparable CD3 expression, with no significant difference between them (*p* = 0.59). CD20 levels were higher in nonaggressive CGCGs but did not reach statistical significance (Mann–Whitney U test; *p* = 0.07). Mast cell density was significantly lower in PGCGs compared with intraosseous nonaggressive CGCGs. **Conclusions**: The present study shows that GCTs of bone, CGCGs, and PGCGs possess distinct immune microenvironmental profiles. Aggressive lesions demonstrate reduced lymphocyte infiltration and increased mast cell density, a pattern particularly evident in GCTs of bone. This imbalance may contribute to their aggressive behavior by enabling them to escape host immune regulation.

## 1. Introduction

Giant cell lesions (GCLs) share similar histopathologic features characterized by the presence of numerous osteoclast-like multinucleated giant cells (MGCs) in a background of mononuclear mesenchymal cells. In the maxillofacial region, giant cell granulomas have varied clinical manifestations and unpredictable biological behaviors [1]. Central giant cell granuloma (CGCG) is a rare, localized, and benign intraosseous lesion that is categorized into nonaggressive and aggressive subtypes [2]. In contrast to nonaggressive CGCG, aggressive CGCG is characterized by pain, paranesthesia, root resorption, rapid growth, a size of >5 cm, cortical perforation, or recurrence after surgical treatment [2]. It is more common in the second and third decades of life and anterior part of the mandible, with female predilection [3]. A precise predisposing factor is yet to be identified; however, an altered bone microenvironment can be a possible cause [4]. Clinical, radiographic, and biochemical criteria are typically used to distinguish aggressive from nonaggressive giant cell lesions, as histological features alone do not reliably predict tumor behavior [5]. Management of CGCGs typically involves conventional surgical curettage, with or without adjunctive medical therapies such as calcitonin, intralesional corticosteroids, interferon-α, bisphosphonates, or denosumab; more aggressive lesions may require en bloc resection [6]. While peripheral giant cell granuloma (PGCG) is a well-defined extraosseous reactive soft tissue lesion that may be related to local irritating factors or trauma, it can occur over a wide age range, with more frequent occurrence in females [7]. Treatment consists of surgical removal of the lesion with the entire base and its underlying irritants. It recurs if not completely excised [8]. Conversely, giant cell tumor (GCT) of the bone is an aggressive neoplasm. It is associated with a large biological spectrum ranging from a latent benign to a highly recurrent neoplasm, predominantly occurring in young adults aged 20 to 40 [8]. It is characterized by osteolytic activity and carries risks of recurrence and distant metastasis [9]. The typical presentation of GCT of bone is a solitary lesion involving the meta-epiphyseal region of the long bones [10]. Surgery remains the dominant treatment, although systemic inhibition of receptor activator of nuclear factor kappa-B ligand (RANKL) with denosumab has shown value as an adjuvant approach. Its long-term effects, however, are unclear, and lesions typically recur after discontinuation [11].

Studying the immune cell infiltrates (mainly CD3^+^ T and CD20^+^ B lymphocytes) within the microenvironment of a lesion is important since their type, behavior, and spatial distribution (inside the lesion’s core or surrounding stroma) would influence the lesion’s growth. A recent in-depth review highlights the pivotal contribution of lymphocyte activation and sustained immunologic engagement in promoting tumor regression [12].

Immune cells engage in dynamic interactions with stromal cells, the extracellular matrix, and tumor cells, collectively shaping the behavior of the lesion. Within the context of chronic inflammation, their presence can lead to divergent outcomes, either facilitating the elimination of abnormal cells or fostering a microenvironment that supports disease progression [13]. Consistent with previous observations, an in vitro study by Muscolo and Ayerza revealed that lymphocytes are activated upon exposure to human GCT cells. GCT of bone in Stage I has a significantly elevated T-cell expression compared with Stages II and III [14]. In a more recent analysis, researchers identified a significant correlation between the GCT of bone/stroma ratio and immune cell infiltration, particularly CD3^+^ T and CD20^+^ B lymphocytes [15].

The concept of immune surveillance has gained renewed significance, particularly as the presence of tumor-infiltrating lymphocytes has been linked to prognosis in various malignancies. Despite this, many tumors develop effective immune-escape strategies. These include alterations in the human leukocyte antigen (HLA) class I antigen-processing pathway, which impair the proper presentation of tumor-derived peptides to T-cell receptors [16], as well as the suppression of cytotoxic lymphocyte activity through immune-modulatory molecules such as B7-H3. Together, these mechanisms enable cancer cells to evade immune detection and support tumor progression [17].

The involvement of T lymphocytes in oral lesions has been extensively investigated, whereas the role of B cells remains less defined [18]. Recent studies have demonstrated that high levels of CD3^+^ T cell infiltration are frequently observed in well- and moderately differentiated oral squamous cell carcinomas [19]. Complementary findings suggest that CD20^+^ B cells may contribute to anti-tumor immunity [20]. In squamous cell carcinoma of the tongue, elevated levels of both CD3^+^ and CD20^+^ lymphocytes were predictive of better clinical outcomes [20]. Moreover, in salivary gland tumors, both CD3^+^ T cells and CD20^+^ B cells have been identified within intra-tumoral and peripheral regions, with CD3^+^ T cell predominance particularly evident in pleomorphic adenoma and mucoepidermoid carcinoma [21].

Mast cells (MCs) are among the most versatile and rapidly responding cells of the immune system. Within seconds of activation, they release biologically active products [22]. Although MCs are recognized for their role in hypersensitivity reactions, they actively contribute to wound healing, angiogenesis, and host defense against pathogens [23]. Evidence indicates that mast cells are commonly present in various tumors and can be attributed to tumor rejection or tumor promotion [24]. Furthermore, MCs are linked to poor prognosis in various malignancies [25]. Their accumulation in the tumor microenvironment drives carcinogenesis through immunosuppressive mechanisms, angiogenic support, and stimulation of tumor cell proliferation [26]. Increased mast cell density has been associated with worse clinical outcomes in melanoma, adenocarcinomas, squamous cell carcinoma, and Hodgkin lymphoma [26]. Vidal et al. found a significantly high number of MCs in the peri-parenchymal tissue of mucoepidermoid carcinoma, whereas the intra-parenchymal region of pleomorphic adenomas of the minor salivary gland showed the smallest MC count [27]. Farhadi et al. revealed that MC concentrations were higher in CGCGs compared with PGCGs and significantly correlated with VEGF expression and linked it to its aggressive clinical behavior [28].

The current published literature lacks information about the expression of CD3^+^ and CD20^+^ lymphocytes in CGCGs or PGCGs and mast cells in GCT of bone. Since the role of the immune response in the pathogenesis and behaviors of different giant cell lesions remains unclear, the present study aimed to identify the existence and density of CD3^+^ and CD20^+^ lymphocytes and mast cells in these lesions.

## 2. Materials and Methods

This retrospective study was performed between November 2024 and June 2025. It was approved by the scientific committee of the College of Dentistry, University of Sulaimani (EC-24-0052, approval date 16 December 2024). Patients were diagnosed on the basis of clinical, radiographic, and biochemical assessments and subsequently managed at three major hospitals in Sulaimani city. Histopathological evaluations were independently confirmed by two pathologists during the course of clinical care. The available clinicopathological data were obtained from their reports. Patients with aneurysmal bone cysts, systemic diseases such as hyperparathyroidism, or syndromes associated with giant cell lesions (including cherubism and Noonan syndrome) were excluded, as these conditions are known to be linked with giant cell lesions and may represent a distinct pathogenesis from solitary giant cell lesions. Patients were excluded if insufficient tissue and extensive hemorrhage were encountered or if insufficient documentation was available. Ulcerated PGCGs and PGCGs associated with teeth were excluded.

A total of 30 formalin-fixed paraffin-embedded (FFPE) blocks that were previously diagnosed as 10 CGCGs, 10 PGCGs, and 10 GCTs of bone were used. We used the campanacci grading system for benign bone tumors [29]. This system focuses on the clinical behavior of the tumors. Stage I: Intraosseous lesions with well-marginated borders and an intact cortex and inactive lesions. Stage II: More extensive intraosseous lesions associated with a thin cortex without loss of cortical continuity and tumors that are active (show progressive growth and radiologic deformation of the bony cortex) (Stage IIA: without pathological fracture; Stage IIB: with pathological fracture). Stage III: Extraosseous lesions that extend into soft tissue.

For our study, we classified the tumor lesions as either aggressive or nonaggressive [29]. Briefly, for GCT of bone, all campanacci Stage III tumors and any Stage II tumors with pathologic fracture and/or recurrence were considered aggressive (*n* = 5). All other GCTs of bone were categorized as nonaggressive (*n* = 5).

Central giant cell granuloma of the maxillofacial skeleton was characterized as aggressive (*n* = 4) and nonaggressive (*n* = 6) according to the clinical and radiographic criteria defined by Vered et al. [30] in 2025.

Three serial 5 µm sections were cut from each block and mounted on positively charged slides. Two slides underwent immunohistochemical staining for CD3 and CD20 antibodies and were detected by using a biotin-free detection system (Mouse/Rabbit Poly Detector plus DAB HRP Brown, Bio SB™ PI 0265). Sections were deparaffinized in xylene, rehydrated through graded ethanol, and antigen retrieval was performed using citrate buffer (1:100 dilution) at 95–99 °C for 30–60 min in a PT module (epredia™). Endogenous peroxidase was blocked for 10 min, followed by a protein block to prevent nonspecific binding. Slides were incubated with primary antibodies (CD3: Rabbit Monoclonal PI6427; CD20: Mouse Monoclonal PI5195, Bio SB™) for 45 min at 37 °C in a humid chamber. Complement and HRP conjugates were applied sequentially (10 and 15 min, respectively), then sections were treated with DAB and counterstained using Mayer’s hematoxylin.

The third tissue section was stained with toluidine blue. After rehydration, slides underwent sequential staining with Harris’ hematoxylin (70 s), Scott’s bluing solution (1 min), and 0.4% toluidine blue (4 min), followed by rinsing, ethanol (70%) and eosin (5%) dips, dehydration, and permanent mounting.

CD3^+^, CD20^+^, and mast cells were counted in 10 high-power hotspots. Mean counts per case were calculated (mean density). The staining density and the percentage of stained areas in each lesion were assessed and quantified by two investigators at two separate time points. Immune expression density was further classified as low (less than 25+), intermediate (25++ to 75++), or high (greater than 75+++) [27]. Zero expression was considered a negative case [31]. Staining results were determined by quantifying the number of infiltrating stained cells across four high-power fields (40×) within the giant cell lesion sections.

Data were tabulated and analyzed using SPSS V27.0. Means ± SD represent continuous data; percentages were used for categorical variables. Normality was tested via the Shapiro–Wilk test. Parametric data were analyzed with one-way ANOVA and Pearson correlation, and non-parametric data were analyzed with the Kruskal–Wallis test and Spearman correlation. Significance was set at *p* < 0.05.

## 3. Result

There was no significant sex variation among the studied groups; however, PGCG showed a slightly male predilection, and GCT of bone was reported more in females. The mean age of patients with CGCG was significantly younger than in other lesions (27.9 ± 16 years), and PGCG had significantly higher mean age (38.9 ± 18.55 years) (*p* = 0.023). Both CGCG (80%) and PGCG (60%) occurred more in the mandible. On the other hand, 50% of GCTs of bone occurred in the radius bone and to a lesser degree in the tibia (Table 1).

**Figure 1:**
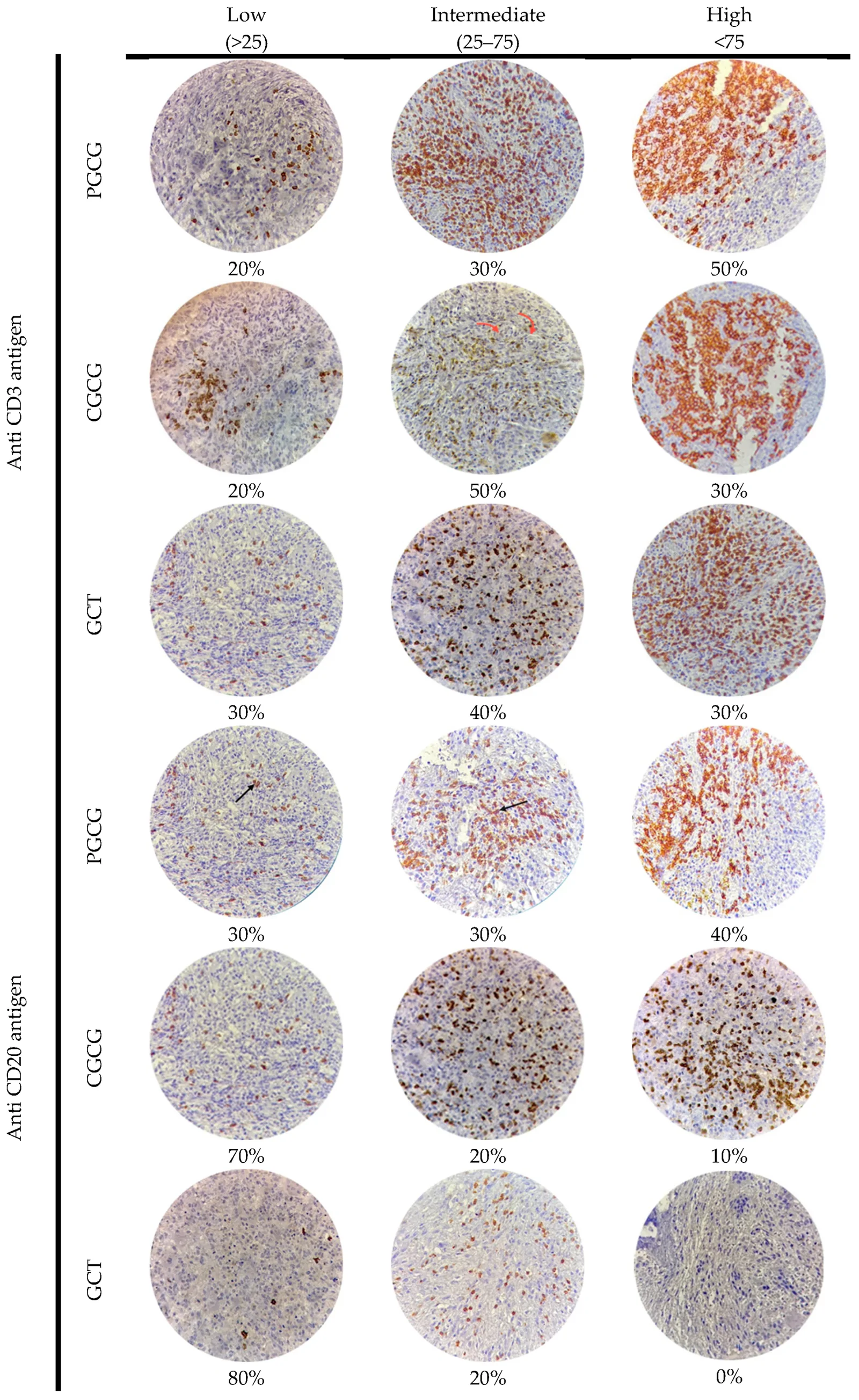
Microphotographs show IHC expression of CD3^+^ and CD20^+^ density in CGCG, PGCG, and GCT. Magnification power ×400. Red arrows indicate CD3 + cells, while black arrows indicate CD20+ cells.

The CD3^+^ expression density was high in 50% of PGCGs and intermediate in 50% of CGCGs, while GCTs of bone did not predominate by a specific density (Figure 1). On the other hand, GCTs of bone and CGCGs both had low CD20^+^ cells, while PGCGs demonstrated 40% CD20^+^ expression at a high score density (Figure 1).

All lesions had CD3^+^ infiltration, but with significant variation in quantification. PGCG showed the highest mean count (69.1 ± 49.3, median = 61.6, IQR = 86.7) and GCT of bone showed the lowest mean count (50.8 ± 29.8, median = 33.7, IQR = 18.6). Thus, CD3^+^ was significantly lower in GCT of bone than in CGCG (*p* = 0.03) and PGCG (*p* < 0.000) (Bonferroni correction post hoc test *p* = 0.0019) (Table 2).

CD20^+^ positivity was very low (mean count < 1) in six cases; five of them were GCTs, and one was a negative case. There was significant variation in CD20^+^ among the three lesions (Kruskal–Wallis test; *p* = 0.001). PGCG had the highest CD20^+^ density expression (mean count = 62.36 ± 58.6, median = 29.7, IQR = 125.1, *p* < 0.001 vs. GCT), followed by CGCG (mean count = 22.7 ± 19.3, median = 15.8, IRQ = 35.6, *p* = 0.01 vs. GCT) and GCT of bone (mean = 2.52 ± 4.3, median = 0.45, IRQ = 3.6) (Bonferroni correction post hoc test; *p* = 0.0014) (Table 2).

**Figure 2:**
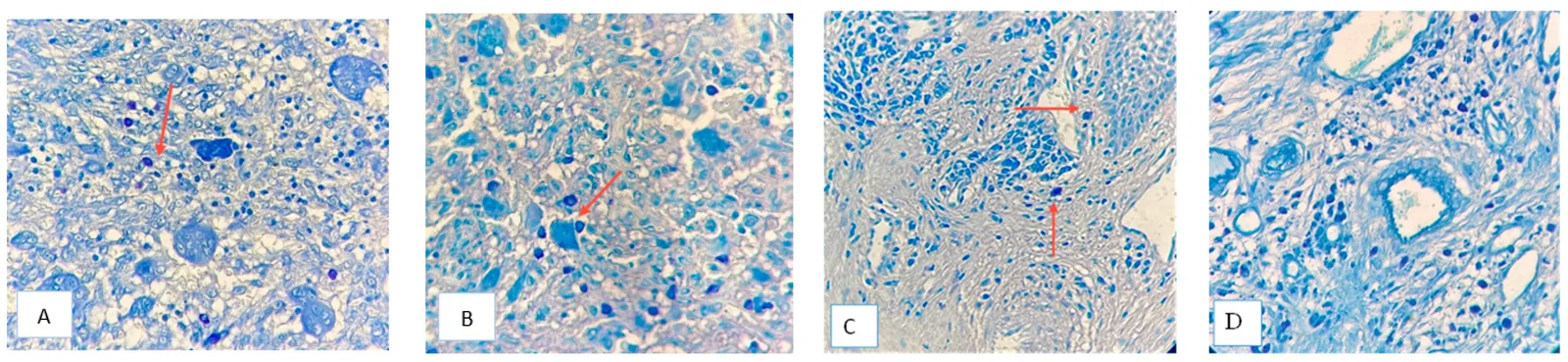
Mast cell aggregation around the giant cell in CGCG (**A**) and GCT of bone (**B**). PGCG shows more MC infiltration around the blood vessel (**C**) and somewhat near the epithelium (**D**). The red arrow shows mast cells as purple and granular mononucleated round-to-ovoid cells for toluidine blue staining ×400.

MCs had significantly different infiltration patterns and densities among the studied lesions. Their aggregation was predominant around giant cells in CGCG and GCT of bone, while they infiltrated around the blood vessel, somewhat near the oral mucosa, in PGCG (one-way ANOVA; *p* = 0.035) (Figure 2, Table 2). PGCG had significantly less mast cell infiltration (mean count 6.3) in comparison with CGCG and GCT of bone (Bonferroni correction post hoc test *p* = 0.04).

**Figure 3:**
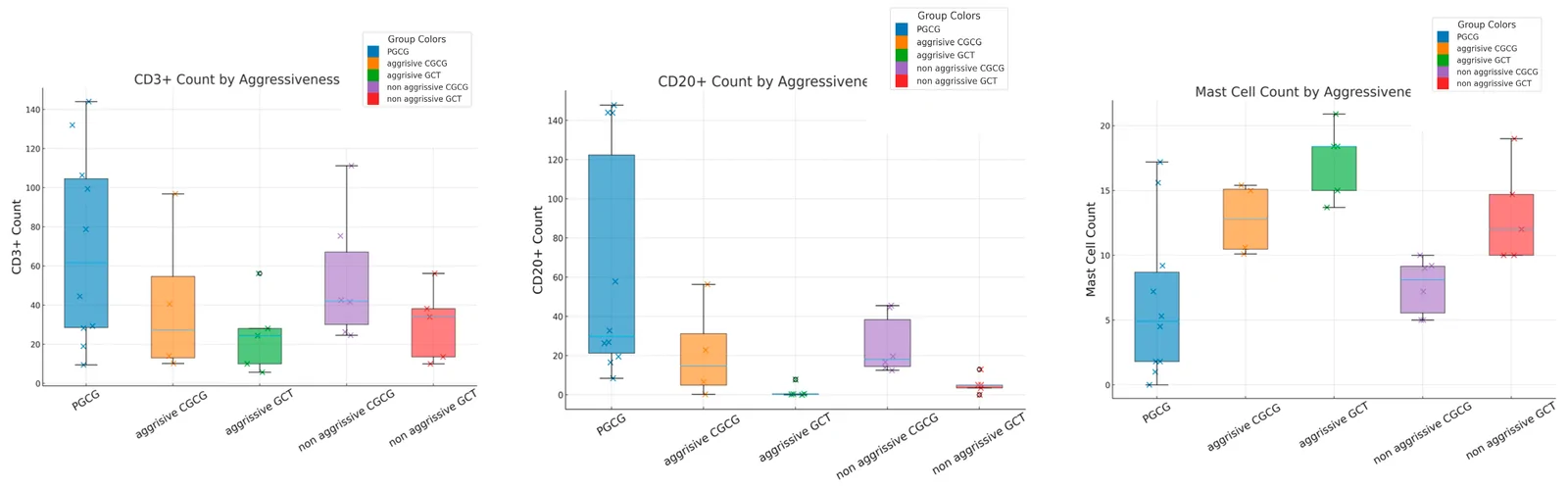
Representative box plots show a comparison of CD3^+^, CD20^+^, and mast cells between aggressive and nonaggressive giant cell lesions. Aggressive lesions show a low number of CD3^+^ and CD20^+^ cells and a high number of mast cells compared with nonaggressive lesions.

Aggressive GCTs showed significantly lower CD3 expression than aggressive CGCGs (Mann–Whitney U test; *p* = 0.003), with no significant difference in CD20 (*p* = 0.53). Mast cell density was markedly higher in aggressive GCTs (Mann–Whitney U test; *p* = 0.001). In nonaggressive lesions, CD3 was significantly higher in nonaggressive CGCGs compared with nonaggressive GCTs (Mann–Whitney U test; *p* = 0.0051), while CD20 (Mann–Whitney U test; *p* = 0.13) and mast cells (Mann–Whitney U test; *p* = 0.62) showed no significant differences. PGCG demonstrated significantly higher CD3 (*p* = 0.0097) and CD20 levels (Mann–Whitney U test; *p* = 0.00069) and lower mast cell density (Mann–Whitney U test; *p* = 0.045) compared with nonaggressive CGCG (Table 3, Figure 3).

A statistically significant and strong positive correlation was observed between CD3^+^ and CD20^+^ in all lesions, indicating a synchronized increase in T-cell and B-cell infiltration. The correlation was strongest in CGCG (r = 0.794, *p* = 0.006), followed by PGCG (r = 0.855, *p* = 0.0016) and GCT (r = 0.726, *p* = 0.017).

**Figure 4:**
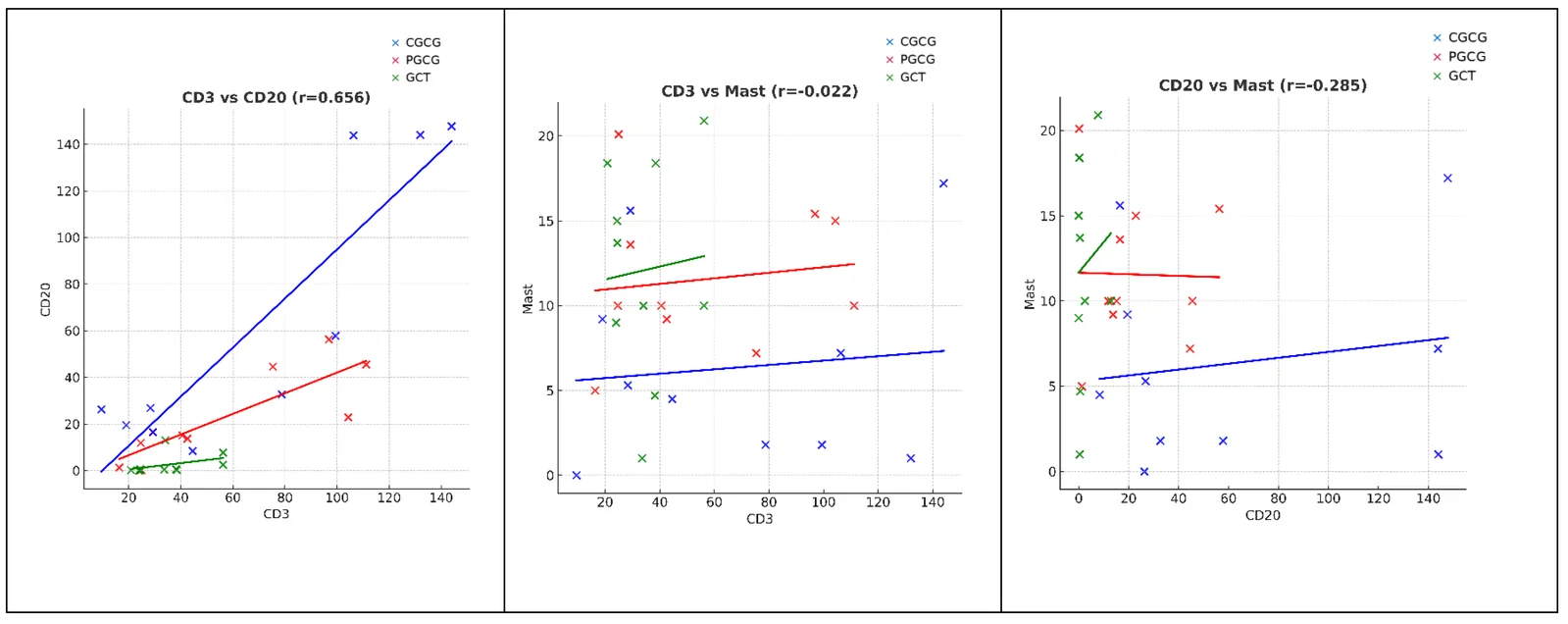
Scatter plots with the regression line demonstrating correlations between CD3^+^, CD20^+^, and mast cells across PGCG, CGCG, and GCT. CD3 and CD20 show a strong positive association, while mast cell density exhibits no measurable correlation with either lymphocytic marker.

In contrast, no significant association was detected between CD3 and mast cell numbers in any of the lesions (PGCG: r = 0.215, *p* = 0.55; CGCG: r = 0.158, *p* = 0.66; GCT: r = 0.107, *p* = 0.77). Similarly, CD20 did not correlate with mast cells in PGCG (r = 0.129, *p* = 0.72), CGCG (r = −0.024, *p* = 0.95), or GCT (r = −0.024, *p* = 0.95) (Figure 4).

## 4. Discussion

In the present study, the peak incidence of GCLs was observed during the second and third decades of life, which is consistent with Badri et al. [32]. CGCGs tend to occur more frequently in younger patients compared with PGCG and GCT of bone. This result is consistent with previous clinicopathological reports [33,34]. A female predominance was noted in GCT patients, while PGCG showed a slight male predilection, aligning with earlier findings [35]. Regarding anatomical distribution, both CGCG and PGCG were more commonly found in the mandible, whereas half of the GCT of bone cases involved the radius bone, similar to what was found in Merza’s study [36].

Furthermore, despite the multiple immunohistochemical, molecular, and morphological studies performed to investigate GCLs of the maxillofacial skeleton and giant cell lesions of bones, there remains a controversy about the pathogenesis and behavior of these lesions.

Over the years, numerous studies have reported diverse approaches to the characterization of GCL types [34,36,37,38]. Building on this background, the present study undertook an immunological analysis to investigate the role of the immune response in the pathogenesis and biological behavior of these lesions. Specifically, it assessed the presence and density of CD3^+^ and CD20^+^ lymphocytes, as well as mast cells, in order to determine whether these immune cell populations may serve as predictors of the clinical course of GCLs. To our knowledge, this is the first study to assess, compare, and correlate the density and distribution of CD3^+^ and CD20^+^ lymphocytes and MCs among GCT of bone, CGCG, and PGCG.

We attempted to analyze central and peripheral giant cell lesions of the maxillofacial region together with giant cell tumors of bone. Although this decision is debated, with some studies suggesting that these entities may be molecularly distinct, the current evidence remains inconclusive. For instance, one investigation reported no detectable histone mutations in central giant cell lesions (*n* = 9) [39], whereas another identified such mutations in 92% of giant cell tumors (49 of 53) [40]. However, these studies are not directly comparable, as the GCT study included separation of stromal and giant cell components prior to sequencing [40], while the CGCG study did not [39]. It is also possible that additional histone alterations would have been revealed with broader genomic analyses, such as whole-genome sequencing.

Moreover, the functional relevance of these histone mutations remains uncertain, as neither study assessed the corresponding protein expression. Considering these limitations, and given the substantial clinical and pathological similarities between CGCG and GCT, particularly in their aggressive variants, we believe that combining these lesions in our analysis is a clinically meaningful approach.

Resnick et al. previously demonstrated that aggressive giant cell lesions of the jaws exhibit a clinical behavior similar to aggressive lesions of the bone, with the exception that a small proportion of giant cell lesions of bone may metastasize [41].

In the jaws, many small, nonaggressive lesions such as peripheral giant cell granulomas (PGCGs) lack an intraosseous niche and are exposed to local intraoral irritative or traumatic factors. In our study, all PGCG cases showing surface ulceration or association with adjacent teeth were excluded. By removing these confounding features, the three lesion groups became more comparable.

There is a renewed interest in studying the role of the immune system in different lesions. Concerning this scope, the immune system’s role in GCLs has yet to be clarified. The immune system plays an active role in determining disease status. T and B lymphocytes can recognize and destroy transformed cells (tumoricidal activity) and halt tumor progression (tumor static role). As a result, the level of these lymphocytes can be used as an indicator of an individual’s immune response to tumors [42]. It is well established that high CD3^+^ correlates with a favorable outcome in oropharyngeal cancer [43].

In our study, both CD3^+^ T cells and CD20^+^ B cells were significantly lower in GCT of bone than in both CGCG and PGCG. A reduction in lymphocytes and duplication of mast cells are related to lesion aggressiveness. Nonaggressive CGCG and PGCG show higher T and B positive cell counts than aggressive lesions of bone. Muscolo and Ayerza indicated that the aggressive behavior of GCT of bone is inversely related to T cell activation [14]. Thus, this tumor can evade immune surveillance [31]. Naji et al. demonstrated higher expression of both types of lymphocytes in PGCG compared with CGCG, which is consistent with the reactive nature of these lesions, in which local irritating factors that trigger an inflammatory response promote a greater release of cytokines such as TNF-α, which may contribute more to angiogenesis than to bone resorption [37].

In the present study, both CD3^+^ and CD20^+^ cells in PGCG were mainly located below the surface epithelium in the perivascular area, while in CGCG and GCT of bone they were mainly seen within the mononuclear stromal cells and near MGCs. Erasha et al. stated that lymphocytes are often found in the perivascular or interstitial areas of the tumor and related to their roles in modulating growth behavior, inflammation, or even osteoclast recruitment [44]. Many researchers believe that the proliferative activity of the GCLs depends on mononuclear stromal cells (fibroblastic/myofibroblastic and endothelial cells) [45,46]. The MGCs carry the osteoclastic phenotype [47] and histiocyte/macrophage markers [48] that may explain the aggregation of lymphocytes around them. On the other hand, aggregation of lymphocytes in perivascular areas and near the surface epithelium may indicate a reactive nature that is associated with a greater release of inflammatory cytokines and increased angiogenesis [49] that recruits monocytic precursors from the blood [50]. The presence of osteoclast-like cells intermingled with mononuclear cells suggests that immune inflammatory mechanisms may participate in the development of PGCG lesions [51].

A few studies have quantified mast cells in reactive oral lesions and reported a significant increase in their count in comparison with normal oral mucosa [52,53,54]. It is believed that MCs share immunopathological mechanisms in oral lesions [53].

Concerning GCLs of the maxillofacial region, MCs infiltrated more in CGCG in comparison with PGCG [28]. This result is consistent with the present work. Furthermore, the current study demonstrated that MCs in GCT of bone were significantly higher than in other lesions but nearly similar to CGCG. No previous work has illustrated this finding before. An adaptive redistribution of MCs within the tumor parenchyma suggests areas that promote tumor invasion and stromal remodeling [27].

The distribution of MCs within the stroma of PGCG, in the vicinity of blood vessels, juxta to the epithelium, may be attributed to the role of endothelial cells and epidermal keratinocytes in synthesizing mast cell growth factor. This key mediator of mast cell migration directs the homing of mast cell precursors beneath the epithelial tissues [55]; in addition, the release of IL-6 and IL-8 by epithelial cells aids in the recruitment of MCs [56].

There is a controversy about MCs’ infiltration and angiogenesis [57]. In the context of reactive oral lesions, the implication is the absence of a relationship between MC count and inflammation and angiogenesis [52]. Thus, MCs have a complex interaction within the microenvironment, and they are contributors to, but not exclusive drivers of, lesion pathogenesis [52].

Many studies suggest a histiocyte/macrophage origin for some of the cellular components and an important role played by stromal MCs in MGC development through the fusion of these cells [47,48]. So, as MGCs form from fused macrophages to remove debris, the aggregation of mast cells around these cells indicates an activated immune response by MGCs [58].

The regulatory impact of MCs on T cell functions is still a controversially debated field. They have been reported both to provoke innate and adaptive immune responses [59] and to have no essential role in the regulation of either CD4^+^ or CD8^+^ T cell immune responses [60,61]. In this study, there was no correlation between mast cell count and CD3^+^ and CD20^+^ cell infiltration. It should be acknowledged that a larger sample size is required to confirm these relations to improve our understanding. Our finding that tissue microenvironment CD20^+^ B cells are strongly correlated with T cells means that activation of T cells contributes to activation of B cells. Immune activation is driven through interactions between immune cells and tumor-associated antigens [62]. T cells activate B cells through a T-cell-dependent process where a B cell internalizes a protein antigen, processes it, and presents it on its surface via MHC class II molecules [62].

Certain limitations were present in this retrospective study that could impact the results. The study’s sample size was constrained by the exclusion of blocks with insufficient tissue and extensive hemorrhage, potentially reducing the statistical power and generalizability. Additionally, the incomplete documentation of clinical features in archived records limited the ability to provide a comprehensive clinical assessment, overall data quality, and completeness.

## 5. Conclusions

The present study highlights the distinct immune profiles of GCT of bone, CGCG, and PGCG. Locally aggressive giant cell lesions are associated with lower lymphocyte infiltration and higher expression of mast cells compared with nonaggressive lesions. The reduced lymphocyte infiltration and higher mast cell counts in GCT of bone may explain its aggressive behavior. These may suggest that the progression and infiltrative behavior of certain giant cell lesions may be facilitated by their ability to evade the host immune response.

## Figures and Tables

**Table 1:** Frequency and percentage distribution of sex, age of patients, and lesion site and behavior in all studied groups.

Study Groups	Sex			Age		Site				Aggressiveness	
	Male *n* (%)	Female *n* (%)	*p* Value	Mean ± SD	*p* Value	Maxilla		Mandible	*p* Value	No.	No.
CGCG	5 (50)	5 (50)	0.315	27.90 ± 16.73	0.023	2 (20)		8 (80)	0.628	6	4
PGCG	7 (70)	3 (30)		38.90 ± 18.55		4 (40)		6 (60)		10	0
						Radius	Femur	Tibia	0.502		
GCT	2 (20)	8 (80)		30.33 ± 7.71		5 (50)	3 (30)	2 (20)		5	5

CGCG: central giant cell granuloma; PGCG: peripheral giant cell granuloma; GCT: giant cell tumor of bone; *n*: number; SD: standard deviation.

**Table 2:** The median count of CD3^+^ and CD20^+^ lymphocytes with the mean mast cell count in the studied giant cell lesions.

Study Groups	CD3+ Cells			CD20+ Cells			Mast Cells			
	Median	IQR	* *p* Value	Median	IQR	*p* Value	Mean ± SD	Mini	Maxi	^$^ *p* Value
CGCG	41.5	73.8	0.03	15.8	35.6	0.001	12.1 ± 5.3	5.00	21.40	0.035
PGCG	61.6	86.7	<0.000	29.7	125.1		6.3 ± 6.0	1.00	17.20	
GCT	33.7	18.6	0.04	0.45	3.6		13.1 ± 6.3	1.00	20.9	

* Kruskal–Wallis test for GCT of bone versus CGCG and PGCG, ^$^ one-way ANOVA. CGCG: central giant cell granuloma; PGCG: peripheral giant cell granuloma; GCT: giant cell tumor of bone; SD: standard deviation; Mini: minimum; Maxi: maximum.

**Table 3:** The median count of CD3^+^ and CD20^+^ lymphocytes with the mean mast cell count according to lesion behavior.

Lesion Behavior	Group	CD3^+^		*p*-Value	CD20^+^		*p*-Value	Mast Cells (Mean ± SD)	*p*-Value
		Median	IQR		Median	IQR			
Nonaggressive	CGCG	58.9	27.7	0.005	18.4	16.7	0.13	12.11 ± 6.36	0.62
	GCT	41.5	36.7		15.75	11.14		16.36 ± 6.03	
Aggressive	CGCG	33.78	11.9	0.003	1.45	3.7	0.053	16.03 ± 2.82	0.001
	GCT	24.35	3.6		0.4	0.3		30.0 ± 6.72	
Nonaggressive	CGCG	58.9	27.7	0.009	18.4	16.7	0.0006	12.11 ± 6.36	0.045
	PGCG	61.6	86.7		29.7	125.1		6.3 ± 6.0	

Mann–Whitney U test for aggressive and nonaggressive lesions; CGCG: central giant cell granuloma; PGCG: peripheral giant cell granuloma; GCT: giant cell tumor of bone; SD: standard deviation; IQR: interquartile range.

## Data Availability

The data presented in this study are available on request from the corresponding author.

## Abbreviations

The following abbreviations are used in this manuscript:

GCLs	Giant cell lesions
CGCG	Central giant cell granuloma
PGCG	Peripheral giant cell granuloma
GCT	Giant cell tumor of long bone
OSSC	Oral squamous cell carcinoma
MGCs	Multinucleated giant cells
MCs	Mast cells
IQR	Interquartile range
